# Genetically programmed changes in transcription of the novel progranulin regulator

**DOI:** 10.1007/s00109-020-01942-7

**Published:** 2020-07-03

**Authors:** Maria Keller, Claudia Gebhardt, Sandra Huth, Dorit Schleinitz, Henrike Heyne, Markus Scholz, Michael Stumvoll, Yvonne Böttcher, Anke Tönjes, Peter Kovacs

**Affiliations:** 1 Helmholtz Institute for Metabolic, Obesity and Vascular Research (HI-MAG) of the Helmholtz Center Munich, University Hospital Leipzig, University of Leipzig, 04103 Leipzig, Germany; 2 Department of Clinical Sciences, Genetic and Molecular Epidemiology Unit, Lund University, Skåne University Hospital Malmö, 20502 Malmö, Sweden; 3 Medical Department III – Endocrinology, Nephrology, Rheumatology, University of Leipzig Medical Center, 04103 Leipzig, Germany; 4 Institute of Biochemistry, Medical Faculty, University of Leipzig, 04103 Leipzig, Germany; 5 Institute of Human Genetics, University of Leipzig, 04103 Leipzig, Germany; 6 Institute for Medical Informatics, Statistics and Epidemiology, University of Leipzig, 04107 Leipzig, Germany; 7 Institute of Clinical Medicine, University of Oslo, Oslo, Norway; 8 Department of Clinical Molecular Biology, Akershus Universitetssykehus, Lørenskog, Norway

**Keywords:** *PSRC1*, Progranulin, DNA methylation, EMSA

## Abstract

**Abstract:**

Progranulin is a glycoprotein marking chronic inflammation in obesity and type 2 diabetes. Previous studies suggested *PSRC1* (proline and serine rich coiled-coil 1) to be a target of genetic variants associated with serum progranulin levels. We aimed to identify potentially functional variants and characterize their role in regulation of *PSRC1*. Phylogenetic module complexity analysis (PMCA) prioritized four polymorphisms (rs12740374, rs629301, rs660240, rs7528419) altering transcription factor binding sites with an overall score for potential regulatory function of *S*_all_ > 7.0. The effects of these variants on transcriptional activity and binding of transcription factors were tested by luciferase reporter and electrophoretic mobility shift assays (EMSA). In parallel, blood DNA promoter methylation of two regions was tested in subjects with a very high (*N* = 100) or a very low (*N* = 100) serum progranulin. Luciferase assays revealed lower activities in vectors carrying the rs629301-A compared with the C allele. Moreover, EMSA indicated a different binding pattern for the two rs629301 alleles, with an additional prominent band for the A allele, which was finally confirmed with the supershift for the Yin Yang 1 transcription factor (YY1). Subjects with high progranulin levels manifested a significantly higher mean DNA methylation (*P* < 1 × 10^−7^) in one promoter region, which was in line with a significantly lower *PSRC1* mRNA expression levels in blood (*P* = 1 × 10^−3^). Consistently, rs629301-A allele was associated with lower *PSRC1* mRNA expression (*P* < 1 × 10^−7^). Our data suggest that the progranulin-associated variant rs629301 modifies the transcription of *PSRC1* through alteration of YY1 binding capacity. DNA methylation studies further support the role of *PSRC1* in regulation of progranulin serum levels.

**Key messages:**

- *PSRC1* (proline and serine rich coiled-coil 1) SNPs are associated with serum progranulin levels.
- rs629301 regulates *PSRC1* expression by affecting Yin Yang 1 transcription factor (YY1) binding.
- *PSRC1* is also epigenetically regulated in subjects with high progranulin levels.

**Electronic supplementary material:**

The online version of this article (10.1007/s00109-020-01942-7) contains supplementary material, which is available to authorized users.

## Introduction

Progranulin (PGRN) is a glycoprotein with a wide range of functions involved, e.g., in inflammatory pathways, metabolism, cell proliferation, and lysosome regulation [1–3]. PGRN is encoded by the *GRN* gene [4], whose mutations can cause frontotemporal lobar degeneration and neuronal ceroid lipofuscinosis [5, 6], but may potentially be also involved in the pathogenesis of Alzheimer’s disease [6–9].

Recent genome-wide association studies identified several loci associated with serum progranulin levels. The most prominent are the *CELSR2-PSRC1-MYBPHL-SORT1*, the *CDH23-PSAP*, and the *GRN* locus [10–12]. In particular, the Sortilin 1 gene (*SORT1*) has been extensively studied as a target gene of the associated variants and shown to regulate circulating low-density lipoprotein levels influencing risk of cardiovascular diseases [13, 14]. *SORT1* is affected by several microRNAs, e.g., miR-146a and miR-182, and is considered to play a role in arterial calcification and chronic inflammation in endothelial cells [15–17].

Besides *SORT1*, *PSRC1* (proline and serine rich coiled-coil 1) has been suggested by recent data including eQTLs (expression quantitative trait locus) [11] to be a target gene of single-nucleotide polymorphisms (SNPs) associated with serum progranulin levels. Furthermore, gene silencing experiments demonstrated the role of *PSRC1* in regulation of progranulin secretion in vitro; however, the functional variant and underlying molecular mechanism have not yet been clarified.

In the present study, we therefore performed in silico and in vitro experiments to identify the potentially causal variants for increased serum progranulin levels and to elucidate their role in transcriptional and epigenetic regulation of *PSRC1.*

## Material and methods

### The Sorbs cohort

A total of 200 individuals with a mean BMI of 26.0 ± 4.2 kg/m^2^ and a mean age of 46 ± 16 years were included in the *PSRC1* promoter methylation analysis. They are part of a metabolically well-characterized cohort of Sorbs from Eastern Germany that was extensively phenotyped for a wide range of anthropometric and metabolic traits (Supplementary Table 1) [18, 19]. The 200 subjects were selected according to their maximal distance in progranulin serum levels, building one group with very high (mean ± SD 151.98 ± 20.86 ng/ml; *N* = 100) and the other one with very low (mean ± SD 73.98 ± 10.04 ng/ml; *N* = 100) concentrations. In addition, both groups were matched for age and BMI by filtering the groups using *t*-statistics, and for gender and smoking status by conducting a chi-square test. Subjects with diabetes were not included in the present analysis. All participants gave their written informed consent, and the study was approved by the ethics committee of the University of Leipzig.

Detailed description of all study participants is provided in the Supplemental Table 1.

### Functional annotation

Functional annotation for all SNPs in linkage disequilibrium (LD, defined as *r*^2^ ≥ 0.86 in Europeans of the 1000 Genomes Phase 1 data) with the leading SNP (rs660240) from the initial GWAS (genome-wide association study) for progranulin serum levels [11] was performed using the phylogenetic module complexity analysis (PMCA) [20] by Genomatix GmbH (München, Germany). This method was used to narrow down potentially causal cis-regulatory variants for further functional analysis in vitro.

### Cell culture

HepG2 and HeLa cell lines (ATCC; Manassas, Virginia) were used for all in vitro studies. Cells were maintained in Dulbeccos Modified Eagle Medium (DMEM; Gibco; 5.56-mM glucose, 1-mM pyruvate, 4-mM L-glutamine, ThermoFisher Scientific, Germany) supplemented with 10% fetal bovine serum (Biochrom GmbH, Germany).

### Preparation of reporter constructs

Single-stranded oligonucleotides harboring each SNP as well as a *Xho* I restriction site on the 5′ and 3′ ends were purchased from MWG-Biotech (Ebersberg, Germany). Oligo-sequences are given in Table 1. Complementary oligos were annealed. Annealed oligos were digested with *Xho* I and cloned to the minimal promotor containing firefly luciferase vector pGL4.23.

**Table 1:** Oligo-sequences used for preparation of reporter constructs and EMSA

	Application	5′-3′ sequence
rs12740374	Luciferase assay	taagcactcgagGAGGAAGAGTAAACACAGTGCTGGCTCGGCTGCCCTGAGG**[G/T]** TGCTCAATCAAGCACAGGTTTCAAGTCTGGGTTCTGGTGTctcgagtgcaag
rs629301	Luciferase assay	taagcactcgagCTAACCATCAGATTGTACAGTTTGGTTGTTGCTGTAAATA[**T/G**] GGTAGCGTTTTGTTGTTGTTGTTTTTTCATGCCCCATACTctcgagtgcaag
rs660240	Luciferase assay	taagcactcgagAGAGAGAGTTAATATATTTGTTTTATTTATTTGCTTTTTG[**T/C**] GTTGGGATGGGTTCGTGTCCAGTCCCGGGGGTCTGATATGctcgagtgcaag
rs7528419	Luciferase assay	taagcactcgagAAAGGACAAAGCCACACGCAGCCAGGGCTTCACACCCTTC[**A/G**] GGCTGCACCCGGGCAGGCCTCAGAACGGTGAGGGGCCAGGctcgagtgcaag
rs12740374	EMSA	CTGCCCTGAGG**[G/T]**TGCTCAATCAAGC
rs629301	EMSA	GCTGTAAATA**[T/G]**GGTAGCGTTTTG
YY1 control	EMSA	CCGATAAGACGCCATTTTAAGTCCTACGTCA

5′-3′ sequences of (+) strand are given. Genomic sequences are depicted in upper case letters; *Xho* I restriction site is given in underlined lower case letters. Parentheses indicate positions of SNP

### Luciferase reporter assays

Functional relevance of candidate SNPs (rs12740374, rs629301, rs660240, rs7528419; all LDs *r*^2^ > 0.94) on transcriptional activity was evaluated by luciferase assay. HepG2 as well as HeLa cells were transfected for the luciferase assay. Cells were co-transfected with luciferase reporter constructs and the Renilla luciferase vector pGL4.74 as internal control using Fugene HD (Promega, Madison, WI) according to the manufacturer’s procedures. After 48 h, cells were harvested and luciferase activity was measured using the Dual Luciferase System (Promega, Madison, WI) as described in the manufacturer’s instructions. Ratio of firefly to Renilla luciferase was calculated. Assays were performed at least in triplicates, and values were normalized to pGL4.23/pGL4.74 empty control.

### Electrophoretic mobility shift assay

The JASPAR (http://jaspar.genereg.net) and PROMO (http://alggen.lsi.upc.es/cgi-bin/promo_v3/promo/promoinit.cgi?dirDB=TF_8.3) online databases were used to predict transcription factor binding to alleles of candidate SNPs. Nuclear protein was extracted from HeLa cells. IRDye 700 (SNPs) or IRDye 800 (positive control) labeled single-stranded oligos were purchased from metabion (Planegg, Germany). Oligo-sequences are given in Table 1. Oligos were annealed to generate double-stranded probes. Electrophoretic mobility shift assay (EMSA) was conducted as follows. Each reaction contained of 7-μg nuclear extract, 4-nmol probe, 1 X binding buffer (10-mM TRIS, 50-mM NaCl, pH 7.5), 2-mM dithiothreitol, 1-μg hering sperm DNA, and 0.25% Tween 20. Binding reaction was performed 30 min at room temperature. For supershift reactions, 4 μg of anti-Yin Yang 1 (YY1) (clone H-10, Santa Cruz Biotechnology Inc., Dallas, TX) was added to the reaction mixture and reactions were incubated for further 30 min. Samples were separated on a 4% native polyacrylamide gel in 0.5 X TRIS-Borate-EDTA (45-mM TRIS, 45-mM boric acid, 1-mM EDTA) followed by visualization with an Odyssey Infrared Imager (LI-COR Biosciences, Lincoln, NE).

### DNA methylation analysis

In parallel, DNA methylation analyses were performed for two sequence segments within the *PSRC1* promoter region (PyroMark assay 1 5′-3′UTR: TCTC**CGCG**CA**CGCG**AGCA**CGCG**CACT**CG**CAGCCTCAACCCT**CG**GCTC**CG**CCAC**CG**GGATGCAGTCTTCTG, PyroMark assay2 5′-3′UTR: AC**CG**TTCTGGAGACTGGGTGCT**CG**G**CG**GCCCAGCAGAGGG AG**CG**GGG) using the pyrosequencing technique as described elsewhere [21] (Qiagen, Hilden, Germany) to test potential epigenetic differences in the *PSRC1* promoter locus between subject with high vs. low progranulin serum levels. PyroMark assays were designed using the PyroMark Assay Design software 2.0 (Qiagen, Hilden, Germany). Briefly, target region was virtually bisulfite converted and primers were selected according to program-specific quality criteria. All reactions were performed in duplicates including two non-template controls per plate and sequenced on PyroMark Q24 (Qiagen, Hilden, Germany).

### rs629301 genotype data

Based on i) our previous GWAS [11] which revealed rs629301 (A>C; MAF = 0.24) to be associated with serum progranulin and as a strong eQTL for the *PSRC1* gene locus, and ii) present findings from the functional annotation studies using PMCA (see below in the “Results” section) further supporting the functional role of this variant, we focused on rs629301 for downstream functional analysis. Genotype data was available from a genome-wide data set for 977 individuals from the German population of Sorbs [22]. Rs629301 genotype distribution (AA/AC/CC: 616/322/39) was in Hardy-Weinberg equilibrium (*P* = 0.7).

### Statistical analysis

CpG methylation levels per site were used as continuous variables, and mean levels were calculated per assay. All analyses were performed using R [23]. Wilcoxon rank sum *t* test was used to compare differences between subjects showing high vs. low progranulin serum levels. A chi-square test was used to estimate differences in the genotype distribution between both groups. Linear regression was applied to detect differences of *PSRC1* mRNA expression levels (available from previous studies) [11] between the different genotypes using additive mode of inheritance (AA vs. AC vs. CC coded as 0 vs. 1 vs. 2, respectively) including adjustments for age and sex.

Statistical analyses for in vitro experiments were performed using the Graphpad Prism software version 6 (Graphpad Software Inc., San Diego, CA). Differences in luciferase assays were assessed by one-way ANOVA followed by Dunnett’s post hoc test to account for multiple comparisons. A *P* value of < 0.05 was considered as statistically significant in all analyses.

## Results

### Phylogenetic module complexity analysis identifies four genetic variants with potential regulatory function

Ten SNPs in tight LD (defined as *r*^2^ ≥ 0.86 in Europeans; Supplementary Table 3) with the GWAS lead SNP rs629301, previously shown to be associated with progranulin serum level [11], were functionally annotated using the PMCA [20] method to identify cis-regulatory variants, most likely affecting *PSRC1* gene expression (Table 2). Among the 10 analyzed variants, we identified 4 SNPs (rs12740374, rs629301, rs660240, and rs7528419) with an overall score for the prediction of a potential regulatory region of *S*_all_ > 7.0, suggesting that these variants are classified and belong to a complex region [20]. The highest PMCA score was found for rs12740374 (*S*_all_ = 9.0, Table 1).

**Table 2:** Phylogenetic module complexity analysis (PMCA) identifies four genetic variants with potential regulatory function

SNP	chr	From	To	#seq	Restricted set of input sequences							Complete set of input sequences							Complex
					*W*_TFBS-restr_	*P*-est *W*_TFBS-restr_	*W*_modules-restr_	*P*-est *W*_module-restr_	*W*_TFBSs in modules-restr_	*P*-est *W*_TFBS in module-restr_	Overall score *S*_all-restr_	*W*_TFBS_	*P*-est *W*_TFBS_	*W*_modules_	*P*-est *W*_module_	*W*_TFBSs in modules_	*P*-est *W*_TFBS in module_	Overall score *S*_all_	
rs12740374	1	109817531	109817650	11	62	0.001	26	0.001	17	0.001	9.0013	227	0.001	81596	0.001	722	0.001	9.0013	1
rs1277930	1	109822084	109822203	10	31	0.167	0	1	0	1	0.7773	100	0.241	9330	0.001	226	0.004	6.0164	0
rs4970836	1	109821738	109821857	6	9	0.568	0	1	0	1	0.2457	18	0.74	88	0.006	40	0.021	4.0304	0
rs583104	1	109821248	109821367	10	54	0.106	27	0.001	23	0.003	6.4976	177	0.133	105319	0.001	747	0.001	6.877	0
rs599839	1	109822107	109822226	9	31	0.71	3	0.366	6	0.337	1.0576	93	0.831	2601	0.076	200	0.321	1.6931	0
rs602633	1	109821452	109821571	7	29	0.051	90	0.001	47	0.001	7.2933	87	0.101	119261	0.001	506	0.001	6.9965	0
rs629301	1	109818247	109818366	11	81	0.001	6180	0.001	336	0.001	9.0013	178	0.019	116072	0.001	1145	0.001	7.7221	1
rs646776	1	109818471	109818590	10	4	0.23	0	1	0	1	0.6383	49	0.116	44618	0.001	212	0.001	6.9364	0
rs660240	1	109817779	109817898	10	109	0.006	8505	0.001	484	0.001	8.2227	195	0.046	113309	0.001	1192	0.001	7.3381	1
rs7528419	1	109817133	109817252	11	31	0.001	27	0.001	26	0.001	9.0013	88	0.014	11687	0.001	333	0.001	7.8547	1

### rs629301 alters gene expression in vitro

**Fig. 1:**
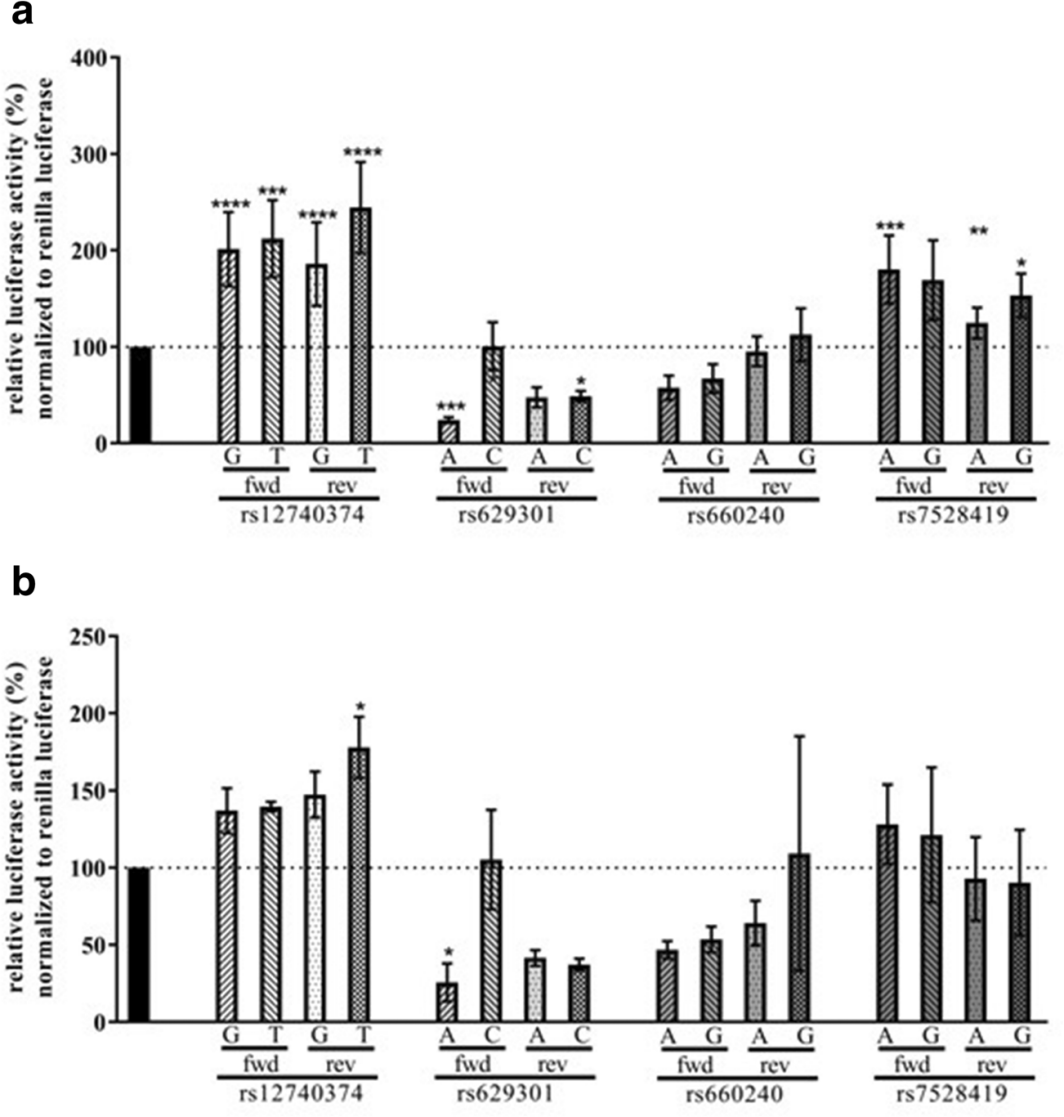
Transcription activity of four candidate SNPs using luciferase assay. Promotor activity of firefly luciferase levels is shown relative to Renilla luciferase. Values are normalized to the luciferase levels of pGL4.23/pGL4.74 empty vector. Three replicate experiments were performed in duplicates, mean ± SD is shown. Analysis was done by one-way ANOVA followed by Dunnett’s post hoc Test.*P < 0.05, **P < 0.01, ***P < 0.001, ***P < 0.0001. ns, not significant. **a** Luciferase activity in HepG2 cells. **b** Luciferase activity in HeLa cells

Functional relevance of the 4 candidate SNPs (rs12740374, rs629301, rs660240, rs7528419) on transcriptional activity was tested by using luciferase reporter assays. We observed significant differences in luciferase activities in both HepG2 and HeLa cells only for rs629301. Luciferase activities were lower in pGL4-rs629301A compared with pGL4-rs629301C allele carrying vector (Fig. 1 a and b). In addition, for rs12740374, the pGL4-rs12740374T showed higher luciferase activity than the pGL4-rs12740374G vector, although both alleles appeared to increase luciferase activities when compared with control vectors (Fig. 1 a and b).

### rs629301 affects binding of Yin Yang 1 regulating the transcription of *PSRC1*

**Fig. 2:**
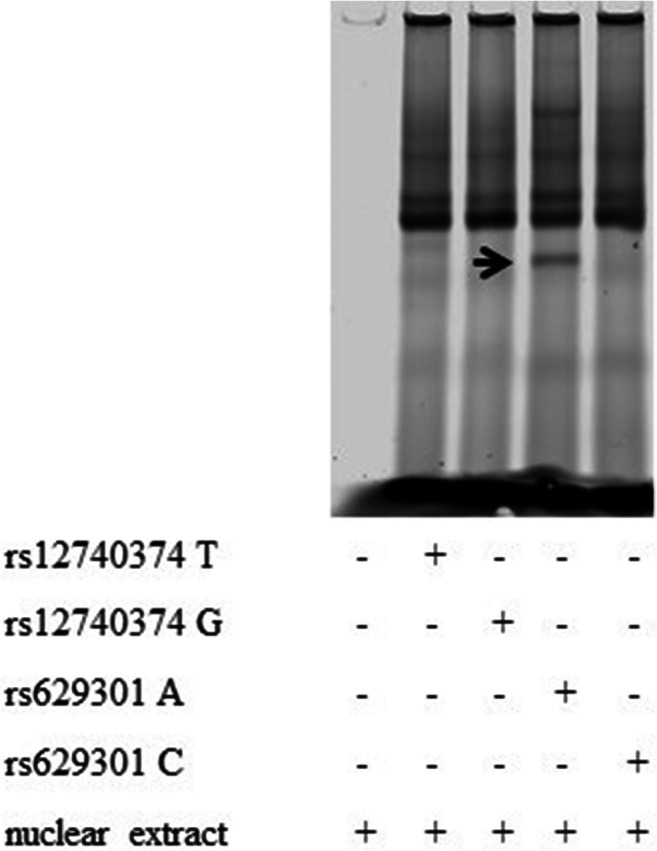
Electrophoretic mobility shift assay (EMSA) of candidate SNPs. Allelic differences in protein binding are only observed for SNP rs629301. Mobility shift is indicated by arrow

**Fig. 3:**
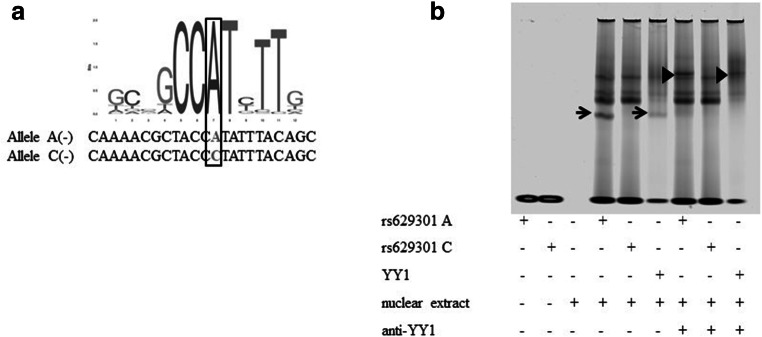
Electrophoretic mobility shift assay (EMSA) of rs629301. **a** A allele of a YY1 binding site. **b** Supershift experiment confirms YY1 binding site prediction. Mobility shift is indicated by arrowheads

Based on findings from luciferase assays, we next tested the binding of the rs629301 and rs12740374 minor and major alleles and demonstrated DNA-protein complexes only using oligo (A) for the major allele sequence of the rs629301 (Fig. 2). In summary, similar protein-binding pattern for the two rs12740374 alleles was observed (Fig. 2). In contrast, a different binding pattern for the two rs629301 alleles was found, with an additional prominent band for the A allele (Fig. 2). Moreover, JASPAR and PROMO databases suggested YY1 transcription factor consensus binding site around rs629301 (Fig. 3a). Addition of YY1 antibody led to a supershift of the prominent band observed in the rs629301 A allele (Fig. 3b). No supershifting for binding in the major and minor allele sequences of rs12740374 (data not shown) and minor allele of the rs629301 was present (Fig. 3b).

### *PSRC1* DNA methylation is increased in subjects with high serum progranulin levels

**Fig. 4:**
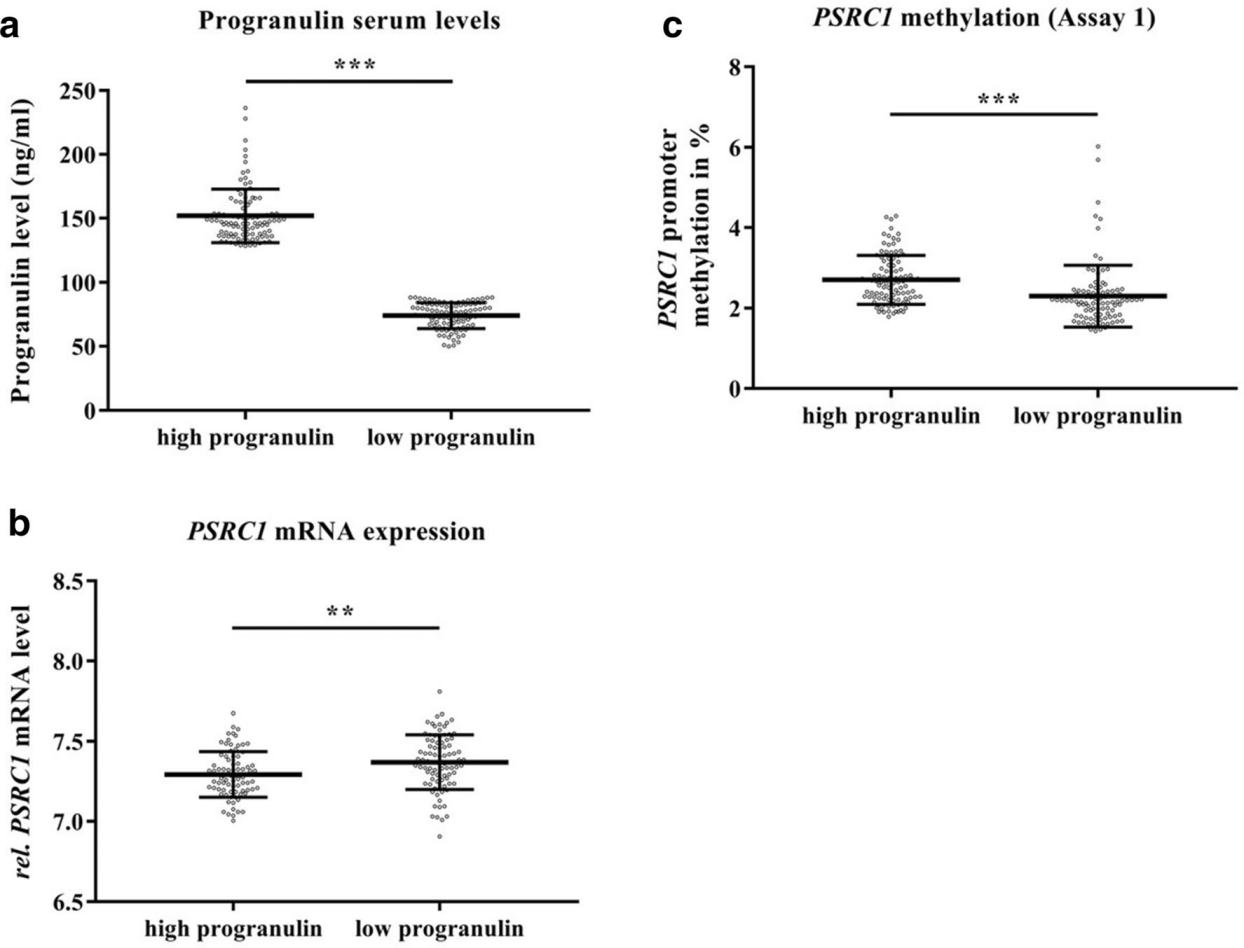
Progranulin levels and epigenetic regulation of PSRC1 in the Sorbs population (*N* = 200). **a** The progranulin serum level in ng/ml. **b** The relative mRNA expression values of PSRC1. **c** The corresponding DNA methylation levels for PSRC1 assay 1 represented in %. All data in **a**–**c** is shown as scatter dot plots representing mean ± SD. **P < 0.01, ***P < 0.001

The subpopulation of individuals selected according to their either very high (*N* = 100, mean ± SD 151.98 ± 20.86 ng/ml) or very low (*N* = 100, mean ± SD 73.98 ± 10.04 ng/ml) serum progranulin level (Fig. 4a, *P* < 1 × 10^−15^) revealed a significant inverse difference in the *PSRC1* mRNA expression levels (Fig. 4b, *P* = 1 × 10^−3^). *PSRC1* promoter methylation for assay 1 (Fig. 4c) is in line with this and shows a significantly (*P* < 1 × 10^−7^) higher mean methylation level in “high progranulin” subjects. The second analyzed assay within the *PSRC1* promoter did not show significant differences between the progranulin groups (data not shown).

### Rs629301 is associated with *PSRC1* mRNA expression and serum progranulin levels

**Fig. 5:**
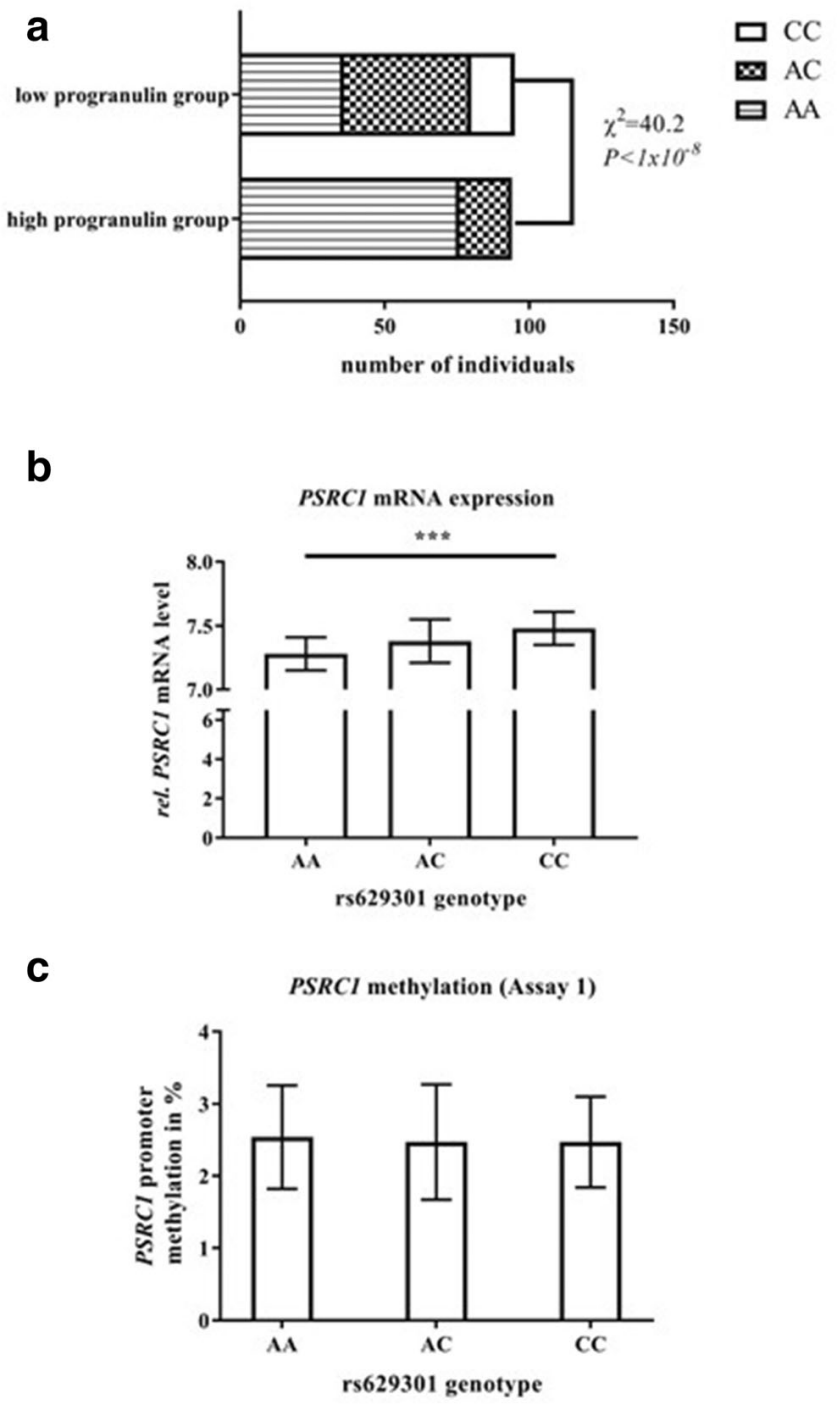
Genotype effects on progranulin and PSRC1 regulation in the Sorbs population (*N* = 200). **a** The genotype distribution between subjects showing a high (AA = 76, AC = 18, CC = 0) vs. low (AA = 36, AC = 44, CC = 15) progranulin serum level. **b** Relative mRNA expression values distributed over the rs629301 genotype. **c** The corresponding DNA methylation levels (%) for PSRC1 assay 1. **a** is presented as number of individuals, and **b** and **c** are presented as bar plots showing mean ± SD values. ***P < 0.001

Distribution of rs629301 genotypes between the progranulin groups (“high” AA = 76, AC = 18, CC = 0; “low” AA = 36; AC = 44; CC = 14) clearly indicated overrepresentation of the A allele in the “high” progranulin group (Fig. 5a, *P* < 1 × 10^−8^). Again, inverse with the protein levels, the A allele was significantly associated with lower *PSRC1* mRNA expression (Fig. 5b, *P* < 1 × 10^−7^, additive mode of inheritance), whereas, albeit not significant (Fig. 5c), *PSRC1* methylation levels were increased.

## Discussion

Progranulin is a secreted protein with important functions in processes including immune and inflammatory responses, metabolism, and embryonic development [24]. It is assumed to be involved in chronic inflammation in obesity and T2D [1, 25]. Heritability of circulating progranulin levels is estimated to be around 30% [11]. A previous genome-wide association meta-analysis of five European cohorts along with subsequent eQTL analyses in peripheral blood mononuclear cells (PBMCs) pointed to *PSRC1* as a potential target gene of the locus significantly associated with serum progranulin levels [11]. Moreover, functional studies in cell cultures supported the role of *PSRC1* in the regulation of progranulin secretion. In particular, 60% reduction of *PSRC1* expression by siRNA silencing in murine 3T3-L1 preadipocytes resulted in a consecutive reduction in progranulin secretion of approximately 30% [11]. To identify the potentially causal variant altering *PSRC1* expression, we performed in silico and in vitro analyses and tested the effect of epigenetic regulation on progranulin serum levels in vivo. In summary, rs629301 turned out to be the most likely causative variant explaining the association of the abovementioned locus with circulating progranulin levels.

An initial PMCA prioritized four polymorphisms (rs12740374, rs629301, rs660240, rs7528419) potentially altering transcription factor binding sites (all with *S*_all_ > 7.0). The effects of these variants on transcriptional activity were tested by luciferase reporter assays, which revealed lower activities in vectors carrying the rs629301-A compared with the C allele. Moreover, EMSA indicated a different binding pattern for the two rs629301 alleles, with an additional prominent band for the A allele. Publicly available databases JASPAR and PROMO predicted a T allele-specific YY1 transcription factor binding site for this locus, which was subsequently confirmed by EMSA supershift using the respective YY1 antibody. Although these findings cannot explain the recently postulated role of PSRC1 in the control of progranulin secretion, they are definitely supporting the regulatory role of genetic variation in PSRC1 and, thus, are complementing the previously reported rs660240 as an eQTL for PSRC1 mRNA in PBMCs [11]. Furthermore, publicly available data for rs629301 additionally support the role of liver as target tissue for the identified effect on PSRC1 regulation by revealing the strongest eQTL on PSRC1 mRNA expression in liver (*P* < 1 × 10^−33^, Supplementary Table 2). In parallel, DNA promoter methylation of two regions (assay 1: 10CpGs; assay 2: 4CpGs) showed that subjects with high progranulin levels manifested a significantly higher mean DNA methylation in one promoter region (assay 1), which was in line with a significantly lower PSRC1 mRNA expression levels in blood. Consistently, rs629301-A allele was associated with lower PSRC1 mRNA expression and higher DNA methylation.

In summary, our data shed more light on the molecular mechanisms behind the associations of genetic variants with progranulin concentrations. Moreover, they strongly support *PSRC1* as a plausible target gene of these genetic variants. Although the underlying functional mechanism linking progranulin and PSRC1 is not fully understood yet, there is a good evidence that PSRC1 might be involved in progranulin-dependent regulation of the Wnt/ß-catenin signaling pathway [26]. As has previously been shown, ß-catenin is directly regulated by PSRC1 [27]. An increased progranulin serum level may inhibit PSRC1 activity via Wnt binding and thereby lead to reduction of ß-catenin, further turning down ß-catenin-dependent transcription factors such as the TCF/LEF family [28]. Vice versa, PSRC1 might affect progranulin as shown by *PSRC1* silencing experiments in vitro [11]. Whether there is a feedback allowing directional switches in mutual effects between PSRC1 and progranulin remains to be investigated in further studies. Nevertheless, further support for the relationship between PSRC1 and progranulin emerges from reports on progranulin-deficient mice [29] and patients with psoriasis, where progranulin was negatively correlated with ß-catenin expression in psoriatic skin lesions [30]. In addition, an enhanced PSCR1 activity may increase ß-catenin expression, which in turn may inhibit NF-kB expression and thereby lead to an anti-inflammatory potential as demonstrated in apoE^−/−^ mice [31].

## Conclusion

In conclusion, our data suggest that the progranulin-associated variant rs629301 modifies the transcription of *PSRC1* through alteration of YY1 binding capacity. YY1 may act indirectly as progranulin repressor most likely by inhibiting *PSRC1* expression. DNA methylation studies further support the role of *PSRC1* in regulation of progranulin serum levels.

## Electronic supplementary material

ESM 1 (XLSX 20.2 kb)s

## Abbreviations

apoE: apolipoprotein E
*CELSR2*: *cadherin EGF LAG seven-pass g-type receptor* 2
EMSA: electrophoretic mobility shift assays
eQTL: expression quantitative trait loci
GWAS: genome-wide association study
LD: linkage disequilibrium
MAF: minor allele frequency
*MYBPHL*: *myosin binding protein H like*
NF-kB: nuclear factor kappa-light-chain-enhancer of activated B cells
PBMC: peripheral blood mononuclear cell
PMCA: phylogenetic module complexity analysis
*PSRC1*: *proline and serine rich coiled-coil* 1
PGRN/*GRN*: progranulin
SNP: single-nucleotide polymorphism
*SORT1*: *sortilin* 1
TCF/LEF: transcription factor/lymphoid enhancer binding factor
YY1: Yin Yang 1 transcription factor
